# Role of Optical Coherence Tomography Imaging in Predicting Progression of Age-Related Macular Disease: A Survey

**DOI:** 10.3390/diagnostics11122313

**Published:** 2021-12-09

**Authors:** Mohamed Elsharkawy, Mostafa Elrazzaz, Mohammed Ghazal, Marah Alhalabi, Ahmed Soliman, Ali Mahmoud, Eman El-Daydamony, Ahmed Atwan, Aristomenis Thanos, Harpal Singh Sandhu, Guruprasad Giridharan, Ayman El-Baz

**Affiliations:** 1 Bioengineering Department, University of Louisville, Louisville, KY 40292, USA; mohamed.elsharkawy@louisville.edu (M.E.); mgelra01@louisville.edu (M.E.); ahmed.soliman@louisville.edu (A.S.); ahmahm01@louisville.edu (A.M.); harpal.sandhu@gmail.com (H.S.S.); gagiri01@louisville.edu (G.G.); 2 Electrical and Computer Engineering Department, College of Engineering, Abu Dhabi University, Abu Dhabi 59911, United Arab Emirates; mohammed.ghazal@adu.ac.ae (M.G.); marah.alhalabi@adu.ac.ae (M.A.); 3 Faculty of Computers and Information, Mansoura University, Mansoura 35516, Egypt; emane_daydamoni@mans.edu.eg (E.E.-D.); ahmed.atwan@nbu.edu.sa (A.A.); 4 Legacy Devers Eye Institute, Portland, OR 97210, USA; athanos@lhs.org

**Keywords:** optical coherence tomography (OCT), computer-aided diagnostic (CAD), age-related macular degeneration (AMD), dry AMD, wet AMD

## Abstract

In developed countries, age-related macular degeneration (AMD), a retinal disease, is the main cause of vision loss in the elderly. Optical Coherence Tomography (OCT) is currently the gold standard for assessing individuals for initial AMD diagnosis. In this paper, we look at how OCT imaging can be used to diagnose AMD. Our main aim is to examine and compare automated computer-aided diagnostic (CAD) systems for diagnosing and grading of AMD. We provide a brief summary, outlining the main aspects of performance assessment and providing a basis for current research in AMD diagnosis. As a result, the only viable alternative is to prevent AMD and stop both this devastating eye condition and unwanted visual impairment. On the other hand, the grading of AMD is very important in order to detect early AMD and prevent patients from reaching advanced AMD disease. In light of this, we explore the remaining issues with automated systems for AMD detection based on OCT imaging, as well as potential directions for diagnosis and monitoring systems based on OCT imaging and telemedicine applications.

## 1. Introduction

Typically, age-related macular degeneration (AMD) results in vision loss in the central retina, i.e., the macula. This disease appears most commonly, in developed countries, in people aged 50 years or older [1,2]. The macula is an important part in the retina and is required for driving, reading, screen use (e.g., watching TV or using a computer), and performing many other daily activities [3]. Routine eye examinations allow ophthalmologists to recognize early signs of the disease, track its progression, and prescribe treatment when it is warranted. Several computer aided diagnosis (CAD) techniques have been used to monitor and control the process of detecting the AMD disease at the early stages [3,4,5,6,7]. These CAD systems are needed to relieve physicians’ workload.

In a patient with early AMD, drusen, deposits of polymorphous acellular material, build up between Bruch’s membrane and the retinal pigment epithelium (RPE) [8,9]. Additionally, as AMD progresses, alterations in the RPE lead to massive losses of epithelial tissue, a condition known as geographic atrophy (GA). On the other hand, another advanced form of AMD is called wet AMD, and it can cause progressive vision loss. This type of disease is characterized by choroidal neovascularization (or CNV), which is the growth of pathological blood vessels under or into the retina. Type 3 neovascularization is another form of wet AMD and is also known as retinal angiomatous proliferation [10]. As a result, fluid leaks into and beneath the retina (subretinal fluid, or SRF) and/or into the retina itself (intraretinal fluid, or IRF).

There are a wide range of factors that play a role in developing advanced AMD, such as aging, ethnicity, and genetics. AMD is strongly associated with old age [11]. In a study of people aged 43 to 86 years, AMD is three times more likely to occur among those aged 75 and older than among those aged 65 to 74 [12,13]. Additionally, AMD is unusual in young patients. In the U.S., the highest prevalence of AMD is among Caucasians, followed by Hispanics and Asians, while the lowest prevalence is found among African Americans [14]. Those with a family history of the disease are at a greater risk of developing AMD themselves. There are also additional risk indicators for AMD, including abdominal obesity [15], hyperlipidemia [16], hyperopia [17], light iris color [18]; cardiovascular conditions [11], hormonal changes [19], alcohol intake [20], and a low level of vitamin B and D in the blood [21,22].

Currently, different imaging techniques were adopted to detect the changes occurring that denote retinal pathologies associated with AMD. The most significant to emerge in recent years is spectral-domain optical coherence tomography (SD-OCT), which allows for imaging of the retina cross-sectionally. SD-OCT has emerged as a primary tool for retina specialists diagnosing AMD and monitoring its progression, particularly in patients who require treatment [23,24]. SD-OCT has proven invaluable to the diagnosis and management of AMD. It provides detailed, in vivo images of the human macula at resolutions of 5–7 microns for a tissue that itself is normally 250–330 microns thick [25]. Thus, it offers numerous advantages over clinical examination of the fundus and color fundus photographs. OCT can identify subtle amounts of IRF or SRF, early discontinuities of the outer retinal layers or RPE, shallow retinal pigment epithelial detachment (PED), subretinal tissue formation, sub-RPE tissue, RPE rips, and outer retinal tubulations, and it can also measure central macular thickness. It is therefore crucial for retina specialists as well as comprehensive ophthalmologists to interpret OCT images accurately.

OCT operates by projecting low-coherence laser light at an infrared frequency where the retina is partially transparent [26]. The cross-sectional image is reconstructed from the interference pattern of back scattered light. These cross-sectional images of the retina allow the detection of fluids, intraretinal or subretinal tissue, and tissue below the retinal pigment epithelium as well as changes in the retina. By understanding the differences between these phenomena, we are better able to differentiate between the classic membranes, occult membranes, and proliferation of retinal angiomas and disciform scars caused by the disease, as well as facilitate the follow-up of VGEF therapy [27,28,29].

Many surveys on retinal imaging have been conducted in the area of ocular research and AMD diagnosis. In this paper, we provide a very comprehensive review of most studies that investigated the role of OCT and other modalities in AMD diagnosis. In the next section, we illustrate first the different types of AMD in detail, which is divided into two main types: (1) dry AMD and (2) wet AMD.

## 2. Grades of AMD

**Figure 1:**
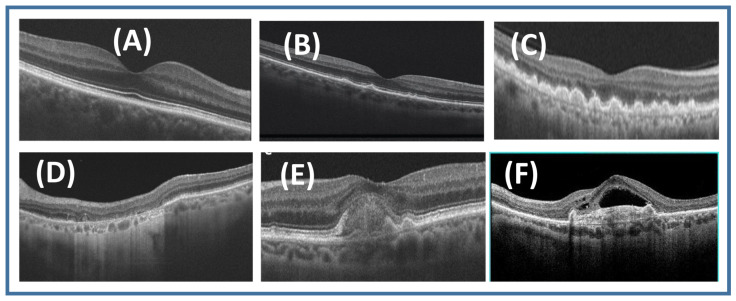
Examples of retinal OCT for (**A**) normal retina, (**B**) early AMD, (**C**) intermediate AMD, (**D**) geographic atrophy (GA), (**E**) inactive wet AMD, and (**F**) active wet AMD.

Clinical classification of AMD is crucial in predicting AMD progression and in developing recommendations for diagnosis, treatment plan and follow up AMD patients. AMD is broadly classified mainly into non-exudative or “dry” type and exudative or “wet” type [30]. Around 85% to 90% of AMD cases are dry. Most dry AMD that reaches an advanced stage leads to atrophy of the RPE; however, a certain percentage of dry AMD may develop into wet AMD with passage of time. Dry type, in contrast to wet type, tends to progress very slowly [31]. The appearance of the macula in various stages of degeneration, with healthy retina for comparison, is shown in Figure 1. In the next subsections, we illustrate each type of AMD and the different categories in each type.

### 2.1. Dry AMD

The difference between dry and wet AMD is that dry AMD does not have any blood or serum leakage. Although dry AMD patients may have loss of vision, they may still have good central vision. However, there are significant functional limitations including limited night vision, fluctuating vision, and difficulty reading due to limited area of central vision. Drusen, or small yellow deposits under the macula, are the main pathological finding in dry AMD [31,32]. Formation of drusen leads to thinning and drying out of the macula, which cause loss of macular function. Dry AMD is classified into “early”, “intermediate”, and “late” according to size of drusen and AMD pigmentary abnormalities [32].

#### 2.1.1. Early Dry AMD

In early AMD, patients may have manifestations related to impaired dark adaptation. They may have difficulty in seeing in dim light or need brighter light. However, most patients do not have any clinical manifestations or vision symptoms in this stage. Ophthalmoscopy often reveals medium-sized drusen (>63 μm and ≤125 μm). No pigmentary abnormalities are seen in early AMD. The risk of progression of early AMD into late AMD within 5–10 years is low [32].

Many studies evaluated for OCT findings that it may detect clinically apparent AMD and found some features that may predict the progression from early to advanced AMD. Ellipsoid zone disruption, drusenoid RPE detachment, RPE thickening, and retinal pigmentary hyperreflective material were significantly associated with high risk of advanced AMD progression. In addition, it was found that some OCT findings are independently associated with higher risk for progression to advanced AMD like total retinal thickness, developing geographic atrophy (GA) features, and choroidal vessel abnormalities [32,33].

#### 2.1.2. Intermediate Dry AMD

In intermediate AMD, the patient has one or more large drusen (≥125 μm) and/or retinal pigment epithelium disturbances that may cause vision loss in one or both eyes. Some patients report symptoms such as blurry spots in the field of vision, difficulty seeing in low light, and contrast sensitivity. Studies of AMD progression have found that within 5 years, 6.3% of patients with intermediate AMD and large drusen in one eye may develop advanced AMD, and the risk increases to 26% if large drusen are found in both eyes [32,33].

#### 2.1.3. Advanced Dry AMD (Geographic Atrophy)

In GA, there are sharply demarcated atrophic lesions of the outer retina, resulting in hypopigmentation due to loss of photoreceptors, or absence of RPE and underlying choriocapillaris in both eyes. It leads to progressive, irreversible loss of visual function. It develops over years, and involves the foveal center late in the disease course. Clinically, it starts with blind spots that appear first in the parafoveal region and then coalesce and enlarge to involve the foveal center, which causes severe central visual loss [33].

An OCT scan of GA shows loss of three outer layers, including the photoreceptors, resulting in thinning of the external band of hyperreflective tissue, and attenuation of RPE/Bruch’s complexes. In addition to detecting hyperreflective foci in the retina overlying drusen and wedge-shaped bands, OCT scans may aid in the detection of changes that precede GA. Atrophic areas show clumps of hyperreflective material, segmented plaques of the outer band with variable reflectivity, and thickened outer hyperreflective bands in OCT images [31].

Optical coherence tomography angiography (OCTA) is informative in cases of GA to visualize atrophy of choriocapillaris beneath the photoreceptors and RPE. This has helped researchers to better understand the development and progression of GA [31,32].

### 2.2. Wet AMD

In wet AMD, the patient may see dark spots in their central vision due to blood or fluid leakage under the macula. Peripheral vision is usually preserved. The main pathogenesis of wet AMD is choroidal neovascularization (CNV) that occurs under the retina and macula. This neovascularization then leaks, causing macular swelling and a reversible loss of vision, or it can bleed, which can be highly toxic to the overlying photoreceptors, sometimes causing irreversible vision loss [33]. In wet AMD, vision loss may be rapid and progressive. Once CNV has developed in one eye, the other eye is at high risk and requires a periodic eye examination. Wet AMD is classified into “classic” and “occult” forms and may be mixed. Furthermore, CNV can be present but inactive, or it can be active, which is characterized by exudation or acute bleeding in the retina. It is always classified as advanced AMD. Usually, it is preceded by dry AMD. The diagnosis is confirmed by OCT. OCT findings should be correlated with the clinical features. It correlates with response to treatment and predicts the success of surgical removal [34].

#### 2.2.1. Inactive Wet AMD

The inactive form is not well demarcated and has less leakage than the active form. In addition, average visual acuity is less impaired, lying between 20/80 and 20/200. The diagnostic criteria for occult CNV are heterogenous hyperfluorescence with late leakage in the macular region associated with PED, stippled hyperfluorescence dots, and deterioration markers [34]. OCT imaging helps in identifying the features of subepithelial occult AMD, the exudative reactions related to it, the presence of PED, and the different changes in the RPE band. In occult inactive AMD, OCT appears as an ill-defined flat lesion with a convex surface [27,31,35].

#### 2.2.2. Active Wet AMD

The active form may appear as a well-defined, highly reflective, fusiform thickening in the subretinal space between the RPE and Bruch’s membrane. It usually results in visual acuity between 20/250 and 20/400, and possibly worse than 20/800, although the use of OCT has increasingly allowed for detection of active wet AMD at earlier stages. OCT can delineate the lesions morphology as well-defined lesion with steep margins and a crater like configuration [34].

## 3. The Image Modalities Used for AMD Classification

**Figure 2:**
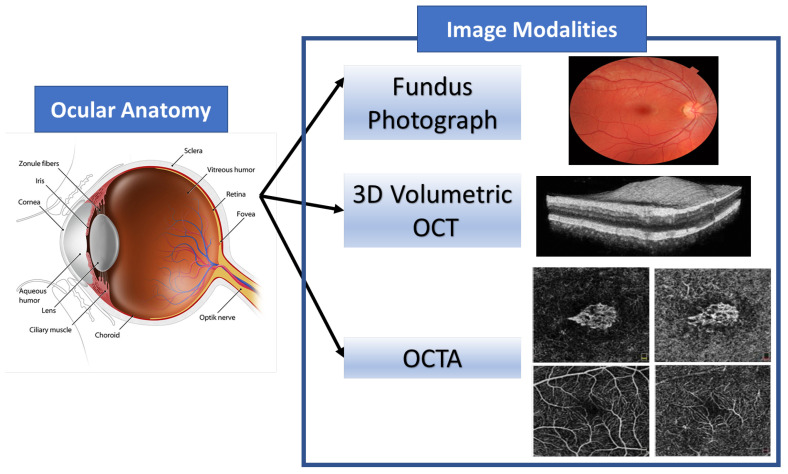
An illustrative structure of ocular anatomy and the images modalities used for eye diagnosis.

**Figure 3:**
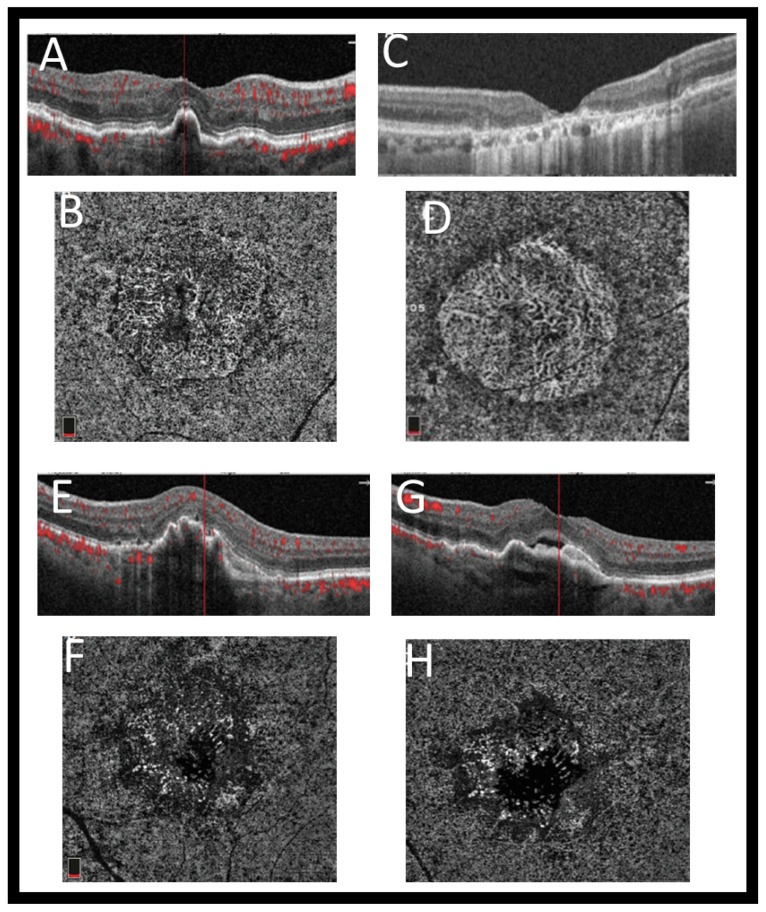
Example of different grades of AMD visualized with Optovue Angiovue OCTA system. The choriocapillaris shows nonexudative macular neovascularization (intermediate AMD), which is mature. On OCT and OCTA, no fluid can be seen (**A**,**B**). (**C**) The tructure SD-OCT can show the area of GA clearly. (**D**) A correlating OCTA scan at choriocapillaris level shows its limitations. In this example, the choriocapillaris has dissolved, exposing the larger choroidal vessels underneath. OCT B-scan (**E**,**G**) of the eye’s choriocapillaris OCTA (**F**,**H**) outlines the presence of immature macular neovascularization and subretinal fluid at different locations of the pigment epithelial detachment.

The eye anatomy is depicted in Figure 2. There are several visible elements of the eye, including the sclera, the cornea, the iris, and the pupil. After moving through the anterior chamber and cornea, the ray of light is scattered by the pupil and lens before finally falling on the retina. The different parts of the eye can be captured by various medical imaging devices. The acquired images are utilized to visualize different pathological findings. There are a number of technologies used to acquire these images. Figure 3 also shows the different image modalities used in diagnosing AMD.

### 3.1. Fundus Image

**Figure 4:**
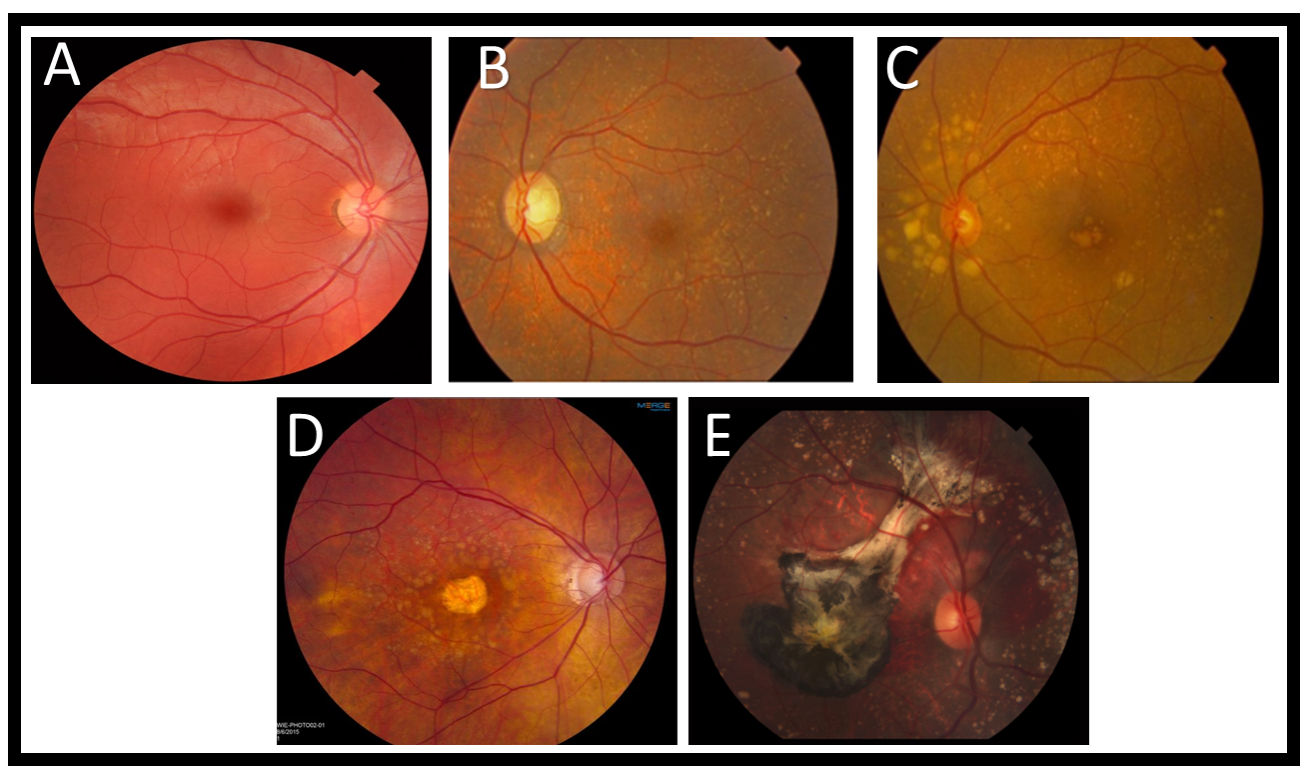
Examples of retinal fundus image for (**A**) an image of a patient with normal retinal health. As a result of more epithelial cells in the macula, it appears darker than other areas in the retina, (**B**) early dry AMD (drusen are deposits under the retina, and this image shows them as yellow. The presence of drusen is a hallmark of AMD), (**C**) One or more large or extensive intermediate drusen, (**D**) advanced dry AMD (it is common for some eyes to develop central atrophy of the RPE and photoreceptors. A significant loss of central vision can result from this, which can be a symptom of advanced dry AMD.), (**E**) wet AMD (this is an example of wet AMD in a retina. An image showing calcified drusen, subretinal bleeding, a black choroidal neovascular membrane (resulting from fibrosis and old blood), and pigmented Xanthophyll in the macula).

The fundus photograph is a visual record that records the appearance of the patient’s retina with an ophthalmoscope. Fundus photography is often required in various eye diseases. Color photography of the fundus has been utilized in the staging and classification of AMD [36]. In early stages of AMD, fundus photography can reveal drusen, which are usually found incidentally during ophthalmoscopy of an asymptomatic individual. Drusen material, which accumulates between RPE and Bruch’s membrane, appears in color fundus photographs as bright white or yellow spots. There are two types: soft spots and hard spots. Hard drusen are characterized by their small size, inconspicuousness, and well-defined, round edges, while soft drusen are less clear and often converge. As drusen commonly occur during normal aging, their presence by itself does not indicate AMD, but as their number and size increase, it increases the risk of AMD with visual symptoms. As AMD progresses, many other signs can be detected, such as pigmentary changes in the RPE that foretell geographic atrophy or exudative abnormalities that indicate conversion to wet AMD [37]. The drusen can be distinguished by the human eye due to their heterogeneous composition that appears as yellowness and brightness in the fundus photographs. However, the recent development of computer algorithms that automatically detect drusen are helpful in differentiating it form other similar pathological appearance, such as hard exudates. Figure 4 shows examples of retinal fundus image for different grades of AMD against normal retina.

### 3.2. Optical Coherence Tomography (OCT)

The optical coherence tomography technique enables in vivo visualization of tissue microstructure via interferometry, and is used to produce cross sectional images of the retina with high resolution. As a medical imaging modality, OCT is most similar to ultrasound, employing light waves in lieu of acoustic waves. Using OCT enables identification of the cellular layers of the retina and can be used to measure the thickness of these layers or the retina as a whole, which helps in the early detection and diagnosis of retinal diseases and conditions. OCT technology has been developed in recent years, and this has made it possible to apply it to a broader range of specialties in medicine [38]. There has been real-time imaging using OCT that occurs at a rate of several frames per second. Developmental biology specimens have recently been analyzed with OCT imaging at the cellular level. Using catheters, endoscopes, and laparoscopes, OCT can provide internal body imaging. One of the significant medical benefits of OCT is its ability to provide cross-sectional imaging of the retina and visualize the microscopic structures in the eye [39].

OCT imaging can be extremely sensitive, which makes it possible to see features with extremely weak backscattering, such as the vitreo-retinal interface, even though the retina has extremely low optical backscattering. RPE and choroid, by contrast, are characterized by their bright (hyperreflective) appearance in OCT images. The layer of retinal nerve fibers, visible on OCT, appears as a hyperreflective structure thickest in the vicinity of the optic disk and getting thinner as it gets closer to the fovea. As well as analyzing dynamic responses of the retina, OCT has been used to study retinal laser injury as well. Analyzing OCT images quantitatively using intelligent algorithms, such as calculating the retinal nerve fiber layer thickness or the thickness of the retinal nerve tissue, can be achieved. OCT is emerging, which can provide quantitative information about which prognostic measurement is used for the diagnosis and monitoring of damage to the retina due to, e.g., glaucoma or diabetic macular edema. Therefore, OCT has been utilized to prevent irreversible loss of vision occur as it detects and diagnoses early stages of disease even before the development of clinical symptoms [40].

### 3.3. Optical Coherence Tomography Angiography (OCTA)

**Figure 5:**
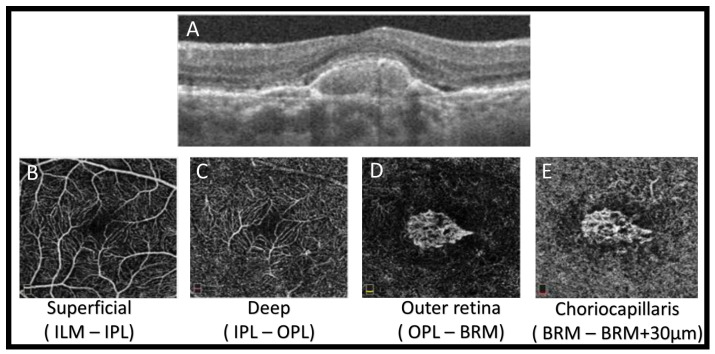
An illustrative OCTA image of a CNV lesion using Optovue Angiovue. We can see the superficial and deep retinal plexuses (**B** and **C**, respectively), as well as the outer retina (**D**) and the choriocapillaries (**E**). An extensive CNV, composed of loops and peripheral anastomoses, is encompassed by a hypointense halo. Subretinal fluid can be seen on SD-OCT (**A**).

In OCTA, instantaneous flow information is captured over a very narrow time window. Like ordinary OCT, it is a non-invasive imaging technique with a wide application in different retinal vascular diseases. It provides volumetric data that specifically localizes and delineates pathology and shows both structural and blood flow information. It helps in diagnosis of different ophthalmologic pathologies by developing a highly detailed view of the retinal vasculature. An OCTA measures the difference between a sequential OCT B-scan and its backscattered signal intensity or amplitude. This is called the decorrelation signal, which requires that the OCT B-scans be taken at precisely the same cross-section in order to construct a map of blood flow. To obtain a densely sampled volume with OCTA, higher imaging speeds are required than are available with most OCT systems at present. Due to the reduced field of view, lower image quality, and greatly increased scanning time, conventional OCT device scanning speeds would have too many trade-offs [41]. Figure 5 shows example of OCTA images of a CNV lesion using Optovue Angiovue. In addition, Figure 3 shows the different grades of AMD, which is visualized with the Optovue Angiovue OCTA system.

## 4. The Abnormalities of AMD

### 4.1. Drusen

Due to apparent RPE undulation, motion artifacts used to evaluate for drusen with OCT is difficult because they show an image that looked like drusen [42,43,44,45]. The introduction of high-speed spectral domain technologies has made determining the size, reflectively, and form of drusen much easier. Due to the varied composition of the underlying substance, small and intermediate-sized drusen may appear as discrete bulges in the RPE with nonuniform reflectivity. Greater elevation of RPE with hyporeflective or moderately reflective substance separating the RPE from the underlying Bruch’s membrane can be noticed in larger drusen [46]. Large drusen are frequently dome-shaped, but as they grow in size, they may become more confluent, with a large lateral dimension and no singular dome-shaped lesion. An OCT study suggests that large confluent drusen appearing in the absence of CNV may be indicative of fluid accumulation under the retina [47]. As the fluid is found between the drusen, its peaks never reach their depressions. This feature may change the management approaches, allowing some patients with subclinical CNV to adopt conservative care with careful follow up and avoid more aggressive treatment with anti-angiogenic treatment. However, more confirmatory research studies are required to address this feature.

Usually in AMD, drusen deposits form between the RPE and the inner (collagenous) edge of Bruch’s membrane. There are variable forms of drusen seen by OCT in different conditions [48]. The Wisconsin age-related maculopathy grading system, introduced in 1991, described reticular drusen as an ill-defined network of broad interlacing ribbons [49]. These were noted as a major risk factor for the development of advanced AMD by the Beaver Dam Eye Study [50]. The introduction of SD-OCT allowed for better characterization of reticular drusen, also known as pseudo-drusen. The term “subretinal drusenoid deposits” was suggested for these drusen, given that they appear as granular hyperreflective material in the subretinal space between the RPE and the IS–OS junctions [51,52].

As a result of Gass’s first description in 1974, a second important variant of drusen was described; weak, yellow, erythematous, round, rounded, sub-RPE lesions in the basal lamina [53,54]. These drusen differ from ordinary drusen in fluorescein angiography, where the variant drusen in the basal lamina show earlier hyperfluorescense compared to usual. Recent histopathologic analysis of these basal laminar drusen suggests that they are otherwise indistinguishable from typical drusen. The term “cuticular” drusen has since been widely adopted to describe them. An OCT examination of cuticular drusen reveals an elevated RPE with rippling of the IS–OS junction [55,56]. A vitelliform lesion may occur with cuticular drusen in the context of early AMD [57,58]. On OCT, vitelliform lesions appear as hyperreflective material localized to the subretinal space, and they mimic the appearance of CNV on fluorescein angiography. Recognition of these lesions is crucial for the proper AMD treatment as they may contain subretinal fluid on OCT due to incomplete RPE phagocytosis of the subretinal material. Numerous studies have demonstrated that drusen size, whether expressed as diameter or area, may be significant prognostic markers for the development of late-stage AMD. Color fundus photographs are ineffective for manual evaluation of drusen and show a poor relationship between graders in a dedicated reading center. Therefore, using SD-OCT for automated quantification and detection of drusen is more promising and fruitful [59,60]. By introducing therapies that target complement pathways, AMD prophylactic interventions can be instituted for extrafoveal GA.

### 4.2. Geographic Atrophy

In GA, dehydration, and calcification of drusen are seen on OCT. There is often confluent RPE atrophy that occurs in conjunction with degradation of the surrounding photoreceptors and choriocapillaris (observable on fluorescein angiography) [61,62]. OCT demonstrates areas of markedly hyperreflective choroidal tissue due to the absence of the overlying RPE [63]. If GA is associated with retinal atrophy, thinning or loss of the outer nuclear layer and the absence of ELM and IS–OS junctions may be seen in OCT. Some hypereflective, drusenoid material may be observed at the level of the RPE, despite preserved outer retina. Variable dynamic changes may indicate imminent atrophy. It is possible to sometimes see a mild swelling of the retina in areas of fovea sparing. Neuronal cellular elements may be predisposed to atrophy by this swelling, which represents a pre-apoptotic stage. Cyst-like spaces, without macular edema, may be present within the inner nuclear layer. In OCT, some changes may be seen in the junctional zone on the margin of the atrophied area. Proximity of outer plexiform layer to Bruch’s membrane indicates that degradation of photoreceptors extends beyond the limit of the GA lesion. It is also possible to see the tapering of the ELM and IS–OS junctions. Pigment migration and alterations in drusen height may also be seen. There may be a link between junctional zone changes and GA pathogenesis and the relative roles of RPE and photoreceptors. In recent studies, OCT images were registered to fundus photos, and the boundaries of GA were delineated using imaging software [64,65]. According to a recent study, patients with a quickly developing form of GA have shown separation of the RPE and Bruch’s membrane in these border zones. A clinically evident GA is correlated with a higher signal total in OCT fundus images obtained by SD-OCT devices.

### 4.3. Neovascular Age-Related Macular Degeneration

Understanding the pathogenesis of neovascularization is crucial in the assessment of neovascular AMD by OCT. An abnormal circulation of blood begins from the choroidal circulation in neovascular AMD. In the subretinal or RPE space, abnormal vessels proliferate after passing anteriorly through Bruch’s membrane breaks [66]. They are immature and therefore incompetent vessels. As a result, they cause fluid exudation and hemorrhage. In the case of severe retinal detachment, this leads to the formation of pathologic “compartments” involving the Bruch’s membrane (PED) and the neurosensory retina (serous retinal detachment). There may be significant disorganization of the overlying retinal architecture and loss of RPE and photoreceptors in disciform scars. As a result of the neovascular invasion, extracellular space in the retina is degraded and remodeled significantly, as fibroblasts invade the space and restore the extracellular space [67,68,69].

## 5. Methods

Here, we review and discuss the research and developments on automatic AMD diagnosis through the analysis of three different imaging modalities. There are many algorithms and databases available that have been developed for treating AMD diseases. As part of an evaluation of the work that uses images as data, the major image modalities employed in CAD applications and research areas are reviewed. Here we present statistics regarding image-based research on the AMD disease.

In our survey of the field, using the PubMed database, we examined peer-reviewed articles up until June 2021 and included all relevant papers. In this paper, we introduce a review of articles for AMD diagnosis and progression that have been developed based on ML. The goal of our study is to capture all publications dealing with automated drusen detection, intraretinal, and subretinal fluids, in addition to subretinal tissue and sub-RPE tissue in the OCT image.

### CAD System for AMD Diagnosing Based on Imaging

During the past 100 years, ocular imaging has progressed significantly and has become a very important component of ocular disease management and clinical care in ophthalmology. CAD derived from radiology and medical images has been the subject of substantial, systematic research and development since the early 1980s. A first report was published in 1973 on retinal image analysis, which focused on vessel segmentation [70]. Baudoin et al. [71] developed an image analysis technique to detect lesions due to diabetic retinopathy in 1984. The rapid advance in image processing related to ophthalmology during the past two decades has paved the way for automated diagnosis of several diseases, including DR [72,73], AMD [74], glaucoma [75], and cataract [76]. The diagnostic systems have the potential to be used for large-scale, rapid screening programs, which can save significant resources and ensure compliance without observer fatigue or bias.

In the recent years, different automated techniques have been investigated to classify OCT based on AMD progression using different deep learning and machine learning methods. Deep learning (DL) methods, which are a state-of-the-art technology that achieves promising results, are mainly based on convolutional neural networks (CNN) to simultaneously perform feature detection, using convolutional kernels, and classification using a sequence of fully connected (FC) layers.

Additionally, as another CAD systems used to differentiate between AMD and other non-AMD diseases, AI is a long-standing discipline of computer science that attempts to make computers understand and act in accordance with the environment, including making decisions. Artificial intelligence (AI) based on ML is motivated in particular by how humans learn [77]. Additionally, many ML algorithms look for patterns in training examples and confirm these patterns for subset classification. When new, previously undiscovered data is presented, the algorithm is able to identify what category they belong to. Learning by triggering the algorithm or by using examples from previous examples can be accomplished with feature-based learning (supervised or unsupervised).

Different automated techniques have been investigated to classify OCT findings related to AMD progression using a variety of machine learning methods (see Table 1). For example, in one study [4], three CNN models were used to differentiate between four grades: normal, dry AMD, active wet, and inactive wet. This system used VGG16, ResNet50, and InceptionV3 and achieved accuracies of 91.40%, 90.73%, and 92.67%, respectively. The three CNN already have evidence of their effectiveness in image recognition. Architectures such as these can be customized for enhanced recognition accuracy by adjusting parameters such as batch size, epoch, learning rate, and optimizer. In order to compare the predictions of AI models with the predictions of ophthalmologists based on clinical verification, a confusion matrix was used. The sensitivity for normal, dry AMD, inactive wet AMD, and active wet AMD using InceptionV3 (the best one in result from the three CNN) was 99.38%, 85.64%, 97.11%, and 88.53%, respectively. For the specificity using InceptionV3, the result was 99.70%, 99.57%, 91.82%, and 98.99% for normal, dry AMD, inactive wet AMD, and active wet, respectively. Freerk et al. [3] presented a ML system that distinguished between healthy OCT and four subcategories of AMD, namely early, intermediate, GA, and CNV. For OCT grading, they used the Bag of Words approach to make text categorization. Then, they express the text categorization of OCT image using a histogram of visual word occurrences. Firstly, the Bag of Words approach generate a dictionary using the training set. Then, a dictionary of representative visual words was then created for AMD classification. Each training OCT volume was randomly sampled from M patches from the detected salient regions. Using the data from the OCT scan, these patches were categorized into five sets according to AMD severity. Using the k-means clustering algorithm, each patch is partitioned into k subsets or clusters according to its mean or cluster centroid, with the mean or cluster centroid being a measure of the distance between points on the patch. Their system achieved a specificity of 91.2% and sensitivity of 98.2%. In another study, An et al. [5] introduced a system for three-way classification between healthy retina, active wet AMD, and inactive wet AMD using pretrained VGG16 CNN [5]. First, they used the CNN model to differentiate between normal images and AMD, achieving an accuracy of 99.2%. Then, they classified wet as wet with fluids and wet without fluids with an accuracy of 95.1%. Transfer learning refers to applying a machine learning model developed previously to another task domain. By transferring learned VGG16 models to the classification task, they are able to solve the task. Training included dropouts, data augmentations such as horizontal flips, random rotations, and random shifts. A total of 100 VGG16 models were used in the training phase, and finally, the model achieving the highest AUC was selected. They did not report the sensitivity and specificity, but they use the AUC when they distinguished between normal and AMD and it was 0.999, while the AUC was 0.992 when they distinguished between normal, AMD with fluid, and AMD without fluid. Motozawa et al. [6] introduced a two-tier classification system with both tries built on CNN models. The first CNN model was used to classify OCT images into normal or AMD, and the second CNN model was used to classify AMD images into those with exudative changes (existing fluids) and without exudative changes (with no fluids). A heat map was created with class activation mapping to highlight the images in the classification that the CNN models emphasized. Moreover, the second model of transfer learning and the single CNN model were compared for speed and stability of learning. In order not to degrade the quality of the image, the CNN models were built using a cropped image. In order to determine the original image classification, three cropped images were reassembled into the original image using CNN models. The first model had 99% accuracy, and sensitivity of 100%, specificity of 91.8%; while the second model demonstrated 93.9% accuracy, and sensitivity of 100%, specificity of 91.8%. In another study [7], the pretrained InceptionV3 CNN was used to detect and distinguish between exudative AMD and healthy retina using OCT images. Using the pre-trained Inception-v3 network, image recognition was run using images obtained from ImageNet dataset. A DCNN model was constructed by training its first layers with approximately 1 million similar images divided into approximately 1000 categories (for example, strawberry, zebra, and banana). Thus, they built a classifier to detect exudative AMD as part of their study. In order to solve this problem, they changed the last layer into a deep CNN to work on the OCT images such that it could diagnose the exudative AMD. The average AMD score was 99.7% and the average healthy score was 92.03%. While the sensitivity and specificity were 100% and 92%, respectively. Feng et al. [78] presented a CNN classification method based on pretrained VGG16 to distinguish between normal, drusen, CNV, and diabetic macular edema (DME). Before grading and labeling the images, the images were acquired. The labeled image dataset was later processed using image preprocessing techniques such as image normalization. To avoid overfitting and enhance the ability of the classifier to generalize, they did not perform image denoising. Lastly, a deep transfer learning method (VGG16 CNN) on retinal OCT images was used to predict the output image from the trained VGG-16 network. They changed the last fully connected layer to be more adjustable with the four output classes. Their system achieved an accuracy of 98.6%, but it did not distinguish between the multiple different stages of AMD with significant detail. The model used archives 100% sensitivity and 100% specificity when distinguishing CNV from normal. In addition, the model archives 98.8% sensitivity and 98.8% specificity when distinguishing DME from normal, while it achieves 98.4% sensitivity and 100% specificity when distinguishing drusen from normal. Choroidal neovascularization can itself be active or inactive, which has critical implications for treatment. Similarly, geographic atrophy, intermediate AMD, and early AMD all carry different prognoses. The existing literature suffers from a number of drawbacks: (i) They use the traditional deep learning CNN to differentiate between AMD categories and normal subjects, not based on the medical landmarks (retina abnormalities); (ii) there is no CAD system that distinguishes between normal eyes and all five clinical grades of AMD.

## 6. Discussion and Future Direction

This review specifically addresses the use of machine learning, especially deep learning, for AMD diagnosis in a qualitative and quantitative manner. Machine learning classifiers are capable of high sensitivity and specificity and have great potential in distinguishing AMD from other conditions. AMD diagnosis has shown promise for ML classifiers in terms of diagnostic accuracy. The use of AI has the potential to improve tele-ophthalmology practices, especially in low-resource areas wherever patients may not have immediate access to an ophthalmologist [88]. It is advantageous to use ML classifiers in rural populations because it allows patients to obtain a diagnosis of AMD early, without the need for a clinician to assure the diagnosis, as well as reduce transportation costs for patients and doctors. In addition, ML offers an opportunity for ophthalmology clinics in urban areas to cut down on patient load and improve efficiency [89].

There was no study found in Africa or in the Middle East, despite the fact that all included studies were conducted in Asia, western Europe, and the United States. In areas with a population of more than a million people, these tools may be more needed for AMD detection, as AMD has become one of the leading causes of vision loss in those countries.

Using OCT-derived biomarkers, GA is the most easily detected form of AMD, while intraretinal/subretinal fluid and PED presented the greatest difficulty to machine learning classifiers. A greater diversity of biomarkers may be available from OCT besides drusen and GA. Despite the nearly identical approaches to drusen and GA segmentation for the two biomarkers, there are differences in the techniques used for intra-/subretinal fluid, PED, and CAD tools. A major factor here could be the availability, and accessibility, of image processing techniques for detection of drusen and GA, owing to their relatively uniform appearance in OCT. It is difficult to compare the different algorithms because of a lack of standardization in their assessment of quality, but CAD tools appear to offer the best overall result. In fact, the sole detection of a pathology is less likely to cause errors than its detection and quantification combined.

It would be worthwhile to examine in a future meta analysis how AI algorithms can be used to classify all AMD types including all dry AMD types in addition to wet AMD types. Furthermore, more future meta analysis about how AI algorithms can be used to differentiate between AMD and non-AMD diseases will be helpful for ophthalmologists. There are some types of AMD that are similar/related to non-AMD diseases in the characteristics. For example, GA and a non-AMD disease (i.e., Stargardt disease) have very similar morphological features on OCT. A macular degeneration caused by an inherited genetic mutation, Stargardt’s disease typically presents in the first 30 years of life, and exhibits progressive atrophy of the macula similar to GA followed by AMD [90]. The retina specialists use the patient’s age as a criterion in diagnosing Stargardt disease [91]. It is also helpful to have family history, but this will not always be beneficial because of the development of “de novo” mutations [90]. In addition, the use of other ophthalmic modalities like fundus imaging and OCTA will be helpful with OCT images to make diagnosis in such cases that have the same characteristics in AMD and non-AMD disease. Moreover, international experts have met under the auspices of the Macula Society and developed a standardized nomenclature framework for classifying neovascular AMD and related lesion characteristics [92]. A crucial part of their proposed classification has been made possible by advances in imaging technology, especially OCT imaging and OCT angiography, which have allowed detailed 3-dimensional analyses of the vascular anatomical structure of neovascular AMD lesions. They found that OCT and OCT angiography techniques can offer direct imaging of anatomic features with a precise evaluation of each component, whether or not it is neovascular.

Ultimately, the objective of this survey is to provide an overview of the present state of collective knowledge about AMD, emphasizing advancements that will enable us to improve the treatment and diagnosis of AMD. Experts in this field have contributed their knowledge, expertise, and vision for improving our understanding, diagnosis, and treatment of AMD. Hopefully, the collection in this survey will provide valuable guidance on how to diagnose and manage AMD.

The implications of our results are important for public health and clinical practice. For example, using the AI system in the workplace will assist in the early detection of AMD so that the necessary precautions can be taken to avoid progression to severe AMD later on. Additionally, in remote areas, where qualified ophthalmologists are not always available, AI significantly increases the efficiency of screening for eye disorders.

In addition, it should be noted that this study has limitations. For example, a few included studies were conducted with quite small sample sizes. This may have affected the AI performance reliability. In addition, AMD was defined differently in the relevant studies, but subgroup analyses were conducted despite this.

## 7. Conclusions

In conclusion, automated detection of AMD using OCT derived biomarkers is promising; however, the type and quality of validation methodologies of this technology vary significantly. The majority of the testing is being conducted on preselected individuals only. Clinical researchers and clinicians will benefit greatly from standardized validation procedures, resulting from the development of algorithms for combined, simultaneous analysis of multiple AMD biomarkers.

## Figures and Tables

**Table 1:** Recent applications of machine learning, including deep learning, to computer-assisted diagnosis of age-related macular degeneration from image data.

Study	Methodology	Year	# of Grades	Weakness	# of Images
An et al. [5]	Develop deep learning techniques using OCT images to AMD classification	2019	They are able to differentiate between AMD with fluids and AMD without fluids	They cannot differentiate between all AMD grades	1625
Motozawa et al. [6]	Separate DL methods tailored to active wet AMD and inactive wet AMD	2019	They are able to distinguish between normal cases and active wet AMD in addition to inactive wet AMD.	They cannot identify the early stages of AMD	1621
Treder et al. [7]	Pretrained InceptionV3 DCNN, multiple computational layers are used to process the input image	2018	They are able to differentiate between healthy and exudative AMD cases	Wet AMD can present with no exudation	1112
Lee et al. [79]	A modified VGG19 DCNN with changed the last fully connected layer to be more adjustable with the two output classes	2017	They are able to differentiate between normal and AMD cases	They cannot differentiate between all AMD grades	43,328
Garcia et al. [80]	Combining mathematical Morphology, Image Processing, and a reliable and effective ML model: a Support Vector Machine (SVM)	2019	They differentiated between healthy and AMD with drusen	Drusen have different sizes which indicates that this is early or intermediate AMD	397
Tan et al. [81]	Develop a deep convolutional neural network (CNN) model capable of detecting AMD autonomously and accurately at the earliest stage	2018	They differentiated between dry and wet AMD	Dry AMD can be in either early or intermediate form and wet AMD can be active or inactive	1110
Hwang et al. [4]	Three pretrained CNN (VGG16, InceptionV3, ResNet50) are used for diagnosis and proposed treatment of AMD	2019	They differentiated between healthy, dry, active wet and in active wet AMD	Dry AMD can be in either early or intermediate form or in its advanced form (GA)	35,900
Li et al. [78]	Investigate how deep learning methods built on the VGG-16 network can enhance OCT’s ability to classify AMD and DME	2019	They differentiated between normal, drusen, CNV and DME	CNV can itself be active or inactive and drusen can be in an earlier form or an intermediate form or in an advanced form (GA)	109,312
Burlina et al. [82]	Generational adversarial networks (GAN) trained on Age-Related Eye Disease Study (AREDS) color fundus images	2019	They distinguished between early, intermediate, and Advanced Stage of AMD	CFP cannot identify subtle amounts of IRF or SRF, early discontinuities of the outer retinal layers or RPE	133,821
Srinivasan et al. [83]	Support vector machines based on multiscale histograms of oriented gradient descriptors are employed as feature vectors	2014	They distinguished between healthy, Dry AMD, and DME	They cannot identify wet AMD; in addition, dry AMD can be in either early or intermediate form or in its advanced form (GA)	90
Hassan et al. [84]	CNNs with multilayered structures that perform Delaunay triangulation and morphing to extract nine layers of retina and choroidal tissue along with macular fluids are employed in this fully autonomous system.	2018	They distinguished between normal, dry AMD, and wet AMD	Wet AMD can be active or inactive; in addition, dry AMD can be in either early or intermediate form or in its advanced form (GA)	46,913
Fraccaro et al. [85]	Models for diagnosing AMD involved a combination of white box methodologies such as logistic regression and decision trees and black box methodologies such as SVM, random forests, and AdaBoost	2015	They distinguished between normal, dry AMD, and wet AMD	Wet AMD can be active or inactive; in addition, dry AMD can be in either early or intermediate form or in its advanced form (GA)	974
Liu et al. [86]	Two SVM classifiers are used to train the OCTs to identify three retinal diseases (AMD, macular hole, macular edema)	2011	They were able to distinguish between normal and AMD cases	They cannot identify or grade each type of AMD	326
Burlina et al. [30]	DL and DCNN were utilized to train fundus images to solve 2-class AMD problem (no/early AMD vs. advanced AMD)	2017	They are able to differentiate between early AMD and advanced AMD	They cannot identify the intermediate stage of AMD in addition to the advanced AMD may contain active wet or inactive wet AMD	67,401
Ting et al. [87]	DL system was used to train the OCT images to diagnose three different eye disease(diabetic retinopathy (DR), glaucoma, and AMD)	2017	They are able to identify intermediate AMD according to AREDS grading system	They cannot identify all categories of AMD	108,558

## Data Availability

No data available.
